# Dose-response association of metformin use and risk of age-related macular degeneration among patients with type 2 diabetes mellitus: a population-based study

**DOI:** 10.3389/fphar.2023.1275095

**Published:** 2023-11-22

**Authors:** Kuang-Hua Huang, Ya-Lan Chang, Chiachi Bonnie Lee, Shuo-Yan Gau, Tung-Han Tsai, Ning-Jen Chung, Chien-Ying Lee

**Affiliations:** ^1^ Department of Health Services Administration, China Medical University, Taichung, Taiwan; ^2^ Department of Pharmacology, Chung Shan Medical University, Taichung, Taiwan; ^3^ Department of Pharmacy, Chung Shan Medical University Hospital, Taichung, Taiwan; ^4^ School of Medicine, Chung Shan Medical University, Taichung, Taiwan

**Keywords:** age-related macular degeneration, metformin, type 2 diabetes mellitus, Pharmacoepidemiology, real world evidence (RWE)

## Abstract

**Background:** Recent studies have demonstrated that patients with type 2 diabetes mellitus (T2DM) who receive metformin have a decreased risk of developing age-related macular degeneration (AMD). However, other studies have also suggested that metformin may increase the risk of AMD development. Therefore, this study investigated the association between treatment with metformin and the risk of AMD in patients with T2DM by using Taiwan’ National Health Insurance Research Database.

**Methods:** Patients who received a diagnosis of new-onset T2DM between 2002 and 2013 were enrolled in this study. The patients were divided into patients treated and not treated with metformin to evaluate the risk of AMD after 5 years of follow-up. The logistic regression was used to estimate the risk of AMD associated with the intensity of treatment with metformin.

**Result:** A total of 7 517 patients (103.16 patients per 10,000 people) developed AMD in 5 years after DM diagnosis. After adjusting for the relevant variables, patients with T2DM treated with <5 defined daily dose (DDD)/month of metformin had a lower risk of AMD (odds ratios [OR]: 0.93; 95% confidence interval [CI]: 0.88 0.99). Patients treated with >25 DDD/month of metformin had a higher risk of AMD (OR: 1.39; 95% CI: 1.08-1.78).

**Conclusion:** Metformin use may be associated with a risk of AMD among patients with T2DM in a dose-dependent association manner, with the greater benefit at lower DDD/month. However, higher DDD/month exhibited an increased risk of AMD.

## Introduction

Age-related macular degeneration (AMD) is the major cause of central irreversible blindness or visual loss among patients aged >50 years in developed countries (Chakravarthy et al., 2010). AMD is typically classified into early and late forms. Patients with early AMD are usually asymptomatic, whereas patients in the late stage of AMD may develop severe progressive vision loss. AMD can be categorized into the 2 following clinical types: nonexudative (dry) and exudative (wet) AMD (Fernandes et al., 2022). Incidence rates of AMD lesions increase substantially with age (Mitchell et al., 2002).

The pathogenesis of AMD is complicated and can be associated with several risk factors, including aging, ocular disorders, systemic diseases, cigarette smoking, diet, body mass index, genetic susceptibility, and environmental conditions (Lim et al., 2012; Ersoy et al., 2014). Studies have investigated whether type 2 diabetes mellitus (T2DM) play a role in AMD development and progression. Several studies have found a positive correlation between T2DM and AMD (Nitsch et al., 2008; Topouzis et al., 2009; Chen et al., 2014; He et al., 2018), whereas some other studies expressed no such effect (Fraser-Bell et al., 2008; Xu et al., 2009). In addition, an inverse association was observed in the Age-Related Eye Disease Study (Clemons et al., 2006).

Several retrospective clinical studies demonstrated that metformin may have a potential role in AMD development (Chen et al., 2019; Lee et al., 2019; Blitzer et al., 2021), while active treatment with metformin is associated an increased risk of dry AMD (Eton et al., 2022). In addition, a meta-analysis study show that metformin is not protective against AMD development (Romdhoniyyah et al., 2021). A study reported that treatment with metformin of low-to-moderate doses is associated with a lower risk of AMD, while higher doses of metformin use did not have reduced risk of AMD development (Blitzer et al., 2021). Conflicting data on the association between metformin exposure dosage and the risk of AMD development. Therefore, we conducted a large-scale nationwide study to determine the association between treatment with metformin and the risk of AMD in patients with T2DM by using data from the National Health Insurance Research Database (NHIRD).

## Material and method

### Data source

This study used the Longitudinal Health Insurance Database (LHID) from 2001 to 2018 as the study database provided by the Health and Welfare Data Science Center (HWDC) of the Ministry of Health and Welfare in Taiwan. The LHID encompasses data pertaining to every individual who is registered within Taiwan’s National Health Insurance (NHI) program. The NHI contains health insurance claims data for 99% of Taiwan’s 23 million residents. Disease diagnoses were coded according to the *International Classification of Diseases, 9th Revision, Clinical Modification* (*ICD-9-CM*) and *ICD, 10th Revision, Clinical Modification* (*ICD-10-CM*). The NHIRD can be used to obtain real-world evidence to support clinical decisions and healthcare policy-making (Chang et al., 2017; Hsieh et al., 2019; Lai et al., 2020). Therefore, we used data from the LHID to analyze the dose-response association of metformin use and risk of AMD among T2DM patients in Taiwan.

### Ethics approval

This study was exempted from informed consent because the personal identification data were encrypted and transformed in the LHID. This study protocol was approved by the Central Regional Research Ethics Committee of China Medical University, Taiwan (No. CRREC-109-011).

### Study participants

**FIGURE 1:**
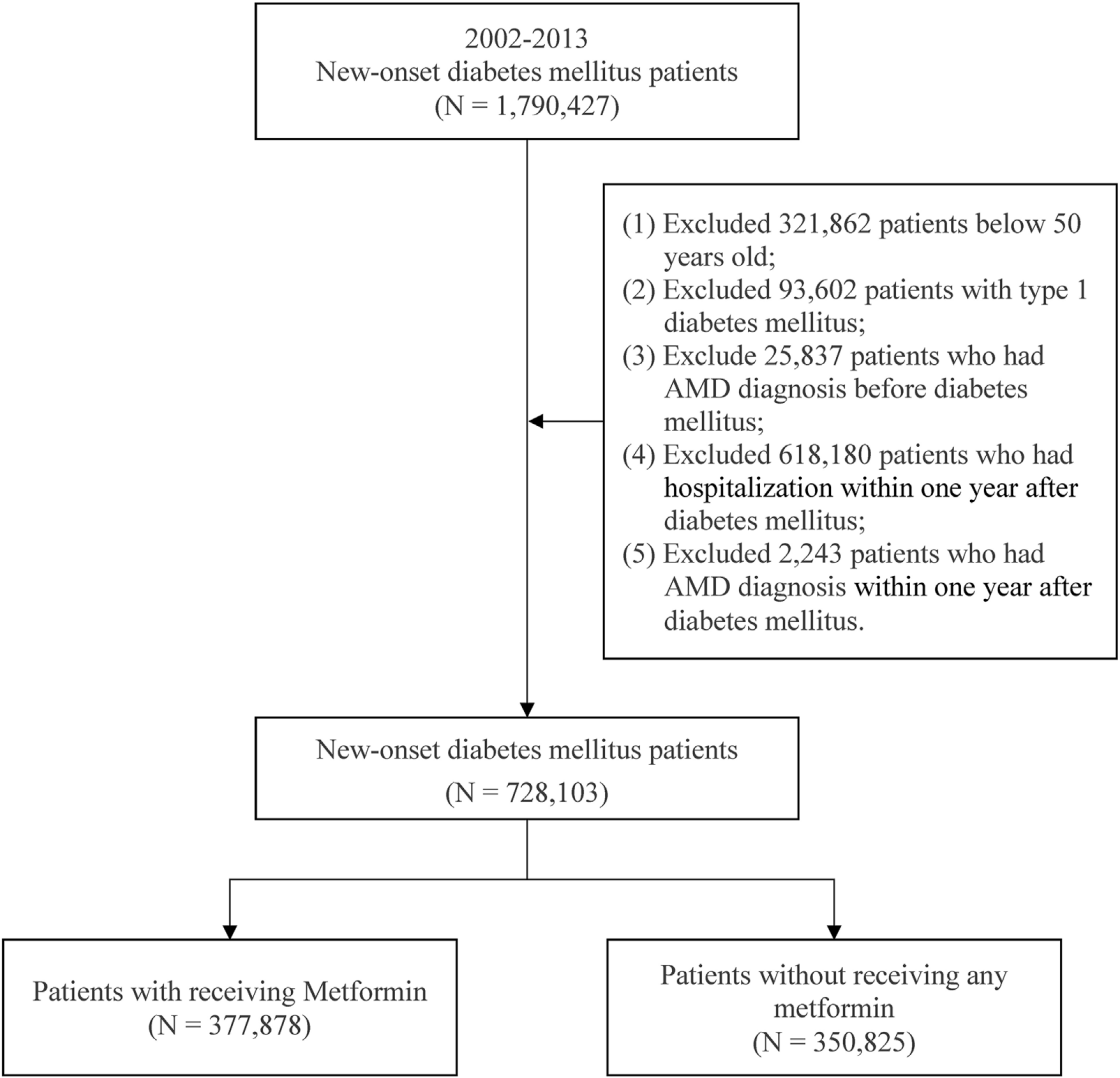
Patient selection process.

This study enrolled patients with new-onset diabetes mellitus (DM) aged ≥50 years from 2002 to 2013. DM (*ICD-9-CM*: 250) was indicated by the presence of 3 outpatient diagnoses. Metformin of the present study was defined according to the Anatomical Therapeutic Chemical (ATC) code A10BA02. The study participant exclusion criteria contained (Chakravarthy et al., 2010) type 1 DM patients, (Fernandes et al., 2022), patients having a diagnosis of AMD before DM, (Mitchell et al., 2002), patients having a diagnosis of AMD in the first year after DM, and (Lim et al., 2012) patients hospitalized within 1 year after DM diagnosis. After selection (Figure 1). There were a total of 728 703 patients with new-onset DM were included in the study. Patients treated with and without metformin were 377 878 patients and 350 825 patients, respectively.

### Study design

This study was a cross-sectional study and used the defined daily dose (DDD) for assessing metformin intake. The DDD is characterized by the World Health Organization as the anticipated average daily maintenance dose for adults. However, the DDD does not necessarily reflect the recommended or prescribed daily dose (Grimmsmann and Himmel, 2011). The DDD of metformin is 2 g (Wellington, 2005), and the observation period prior to treatment with metformin in the present study was 1 year after DM. Based on the study design from previous studies (Chang et al., 2018; Huang et al., 2022a; Huang et al., 2022b), we categorized patients according to the average monthly DDD (expressed as DDD/month) into 5 ranges: 0, <5, 5 15, 15–25, and >25, respectively.

All patients were observed for a 5-year period to investigate the risk of incident AMD. The definition of incident AMD (*ICD-9-CM*: 362.50-362.52, 362.57; *ICD-10-CM*: H35.31-H35.32, H35.36) was indicated by 3 or more outpatient visits within 1 year. Control variables were sex, age, income level, urbanization, diabetes complications severity (DCSI), and AMD-related comorbidities. The DCSI was used to assess the severity of diabetes (Young et al., 2008; Chang et al., 2012). The comorbidities were hyperlipidemia (*ICD-9-CM*: 272.0-272.4), hyperuricemia (*ICD-9-CM*: 790.6), cerebrovascular disease (CVD; ICD-9-CM: 430-438), obesity (*ICD-9-CM*: 278.00), alcoholism (*ICD-9-CM*: 303), nonalcoholic fatty liver disease (NAFLD; *ICD-9-CM*: 571.8), rheumatoid arthritis (RA; *ICD-9-CM*: 714), hypothyroidism (*ICD-9-CM*: 244.9), hepatitis B virus (HBV; *ICD-9-CM*: 070.33), hepatitis C virus (HCV; *ICD-9-CM*: 070.54), sleep disturbance (*ICD-9-CM*: 780), systematic lupus erythematosus (SLE; *ICD-9-CM*: 710.0), chronic kidney disease (CKD; *ICD-9-CM*: 585), migraine (*ICD-9-CM*: 346.90), and hyperthyroidism (*ICD-9-CM*: 242.9).

### Statistical analysis

All analyses were performed using SAS version 9.4 (SAS Institute, Cary, NC, United States). The chi-square test was used to evaluate differences between patients treated with and without metformin. Multiple logistic regression was used to estimate the odds ratios (ORs) and 95% confidence intervals (CIs) for AMD risk after adjustment for sex, age, income level, urbanization, diabetes severity, and comorbidities. All statistical results with *p* < .05 were regarded as statistically significant.

## Results

Table 1 presents the baseline characteristics of all patients. The average age of all patients was 62.06 ± 8.83 years, and 51.42% of all patients were women. Regarding age groups, 23.72% were 50–54 years old, 22.82% were 55–59 years old, 18.00% were 60–64 years old, 13.96% were 65–69 years old, 10.51% were 70–74 years old, and 10.99% were above 75 years old. In patients treated with metformin, the average age was 61.21 ± 8.43 years.

**TABLE 1:** The characteristics of patients with diabetes mellitus.

Variables	Total		Metformin				
			Non-users		Users		*p*-value
	N	%	N	%	N	%	
Total	728,703	100.00	350,825	100.00	377,878	100.00	
Sex							<0.001
Female	374,706	51.42	185,681	52.93	189,025	50.02	
Male	353,997	48.58	165,144	47.07	188,853	49.98	
Age (year)							<0.001
50–54	172,863	23.72	74,579	21.26	98,284	26.01	
55–59	166,290	22.82	74,969	21.37	91,321	24.17	
60–64	131,178	18.00	62,907	17.93	68,271	18.07	
65–69	101,691	13.96	50,466	14.38	51,225	13.56	
70–74	76,584	10.51	40,604	11.57	35,980	9.52	
≥75	80,097	10.99	47,300	13.48	32,797	8.68	
Mean ± SD	62.06 ± 8.83		62.98 ± 9.16		61.21 ± 8.43		
Income level (NTD.)							<0.001
≤21,000	377,872	51.86	186,084	53.04	191,788	50.75	
21,001–33,000	172,793	23.71	77,497	22.09	95,296	25.22	
≥33,001	178,038	24.43	87,244	24.87	90,794	24.03	
Urbanization							<0.001
Level 1	200,346	27.49	102,111	29.11	98,235	26.00	
Level 2	235,727	32.35	112,688	32.12	123,039	32.56	
Level 3	113,396	15.56	52,002	14.82	61,394	16.25	
Level 4	102,480	14.06	48,430	13.80	54,050	14.30	
Level 5	17,112	2.35	8,350	2.38	8,762	2.32	
Level 6	31,238	4.29	14,389	4.10	16,849	4.46	
Level 7	28,404	3.90	12,855	3.66	15,549	4.11	
DCSI score ^a^							<0.001
0	442,189	60.68	209,108	59.60	233,081	61.68	
1	155,131	21.29	74,328	21.19	80,803	21.38	
2+	131,383	18.03	67,389	19.21	63,994	16.94	
Hyperlipidemia							<0.001
No	574,597	78.85	264,377	75.36	310,220	82.10	
Yes	154,106	21.15	86,448	24.64	67,658	17.90	
Hyperuricemia							<0.001
No	722,413	99.14	347,304	99.00	375,109	99.27	
Yes	6,290	0.86	3,521	1.00	2,769	0.73	
Cerebrovascular disease							<0.001
No	690,054	94.70	329,884	94.03	360,170	95.31	
Yes	38,649	5.30	20,941	5.97	17,708	4.69	
Obesity							0.003
No	725,531	99.56	349,382	99.59	376,149	99.54	
Yes	3,172	0.44	1,443	0.41	1,729	0.46	
Alcoholism							0.985
No	728,261	99.94	350,612	99.94	377,649	99.94	
Yes	442	0.06	213	0.06	229	0.06	
NAFLD ^a^							<0.001
No	722,530	99.15	347,661	99.10	374,869	99.20	
Yes	6,173	0.85	3,164	0.90	3,009	0.80	
RA ^a^							<0.001
No	722,471	99.14	347,479	99.05	374,992	99.24	
Yes	6,232	0.86	3,346	0.95	2,886	0.76	
Hypothyroidism							<0.001
No	725,490	99.56	348,848	99.44	376,642	99.67	
Yes	3,213	0.44	1,977	0.56	1,236	0.33	
HBV ^a^							0.299
No	728,574	99.98	350,757	99.98	377,817	99.98	
Yes	129	0.02	68	0.02	61	0.02	
HCV ^c^							<0.001
No	725,443	99.55	349,073	99.50	376,370	99.60	
Yes	3,260	0.45	1,752	0.50	1,508	0.40	
Sleep disturbance							<0.001
No	569,717	78.18	270,541	77.12	299,176	79.17	
Yes	158,986	21.82	80,284	22.88	78,702	20.83	
SLE ^a^							0.024
No	728,319	99.95	350,618	99.94	377,701	99.95	
Yes	384	0.05	207	0.06	177	0.05	
CKD ^a^							<0.001
No	722,880	99.20	346,514	98.77	376,366	99.60	
Yes	5,823	0.80	4,311	1.23	1,512	0.40	
Migraine							0.989
No	725,088	99.50	349,085	99.50	376,003	99.50	
Yes	3,615	0.50	1,740	0.50	1,875	0.50	
Hyperthyroidism							<0.001
No	724,367	99.40	348,106	99.22	376,261	99.57	
Yes	4,336	0.60	2,719	0.78	1,617	0.43	

^a^ Abbreviations: DCSI, diabetes complications severity index; NAFLD, non-alcoholic fatty liver disease; RA, rheumatoid arthritis; HBV, hepatitis B virus; HCV, hepatitis C virus; SLE, systemic lupus erythematosus; CKD.

Table 2 presents the incidence rate per 10,000 people of AMD and the risk of AMD after 5 years of follow-up. Patients not treated with metformin were 350 825 and the incidence rate of AMD was 111.88 patients per 10,000 people; patients treated with metformin <5 DDD/month were 168 198 and the incidence rate of AMD was 94.12 patients per 10,000 people; patients treated with metformin 5–15 DDD/month were 158 992 and the incidence rate of AMD was 95.85 patients per 10,000 people; patients treated with metformin 15–25 DDD/month were 45 478 and the incidence rate of AMD was 93.01 patients per 10,000 people; patients treated with metformin >25 DDD/month were 5210 and the incidence rate of AMD was 119.00 patients per 10,000 people.

**TABLE 2:** Five-year follow-up of incident age-related macular degeneration.

Variables	Five-year follow-up of incident age-related macular degeneration						
	Total N	Events N	Incidence rate per 10,000 people	*p*-value	Adjusted model		
					OR	95% CI	*p*-value
Total	728,703	7,517	103.16				
Intensity of metformin use				<0.001			
Non-users	350,825	3925	111.88		1		
<5	168,198	1583	94.12		0.93	(0.88–0.99)	0.014
5∼15	158,992	1524	95.85		1.00	(0.95–1.07)	0.894
15∼25	45,478	423	93.01		1.01	(0.91–1.12)	0.846
>25	5,210	62	119.00		1.39	(1.08–1.78)	0.011
Sex				0.722			
Female	374,706	3,850	102.75		1		
Male	353,997	3667	103.59		1.08	(1.03–1.13)	0.002
Age (year)				<0.001			
50–54	172,863	580	33.55		1		
55–59	166,290	1010	60.74		1.80	(1.63–2.00)	<0.001
60–64	131,178	1241	94.60		2.79	(2.53–3.08)	<0.001
65–69	101,691	1440	141.61		4.14	(3.75–4.56)	<0.001
70–74	76,584	1455	189.99		5.53	(5.02–6.10)	<0.001
≥75	80,097	1,791	223.60		6.40	(5.82–7.05)	<0.001
Income level (NTD)				<0.001			
≤21,000	377,872	4,498	119.04		1		
21,001–33,000	172,793	1394	80.67		0.83	(0.78–0.88)	<0.001
≥33,001	178,038	1625	91.27		0.93	(0.88–0.99)	0.014
Urbanization				<0.001			
Level 1	200,346	2300	114.80		1		
Level 2	235,727	2335	99.06		0.86	(0.81–0.91)	<0.001
Level 3	113,396	1025	90.39		0.75	(0.69–0.80)	<0.001
Level 4	102,480	1067	104.12		0.78	(0.72–0.84)	<0.001
Level 5	17,112	231	134.99		0.88	(0.77–1.01)	0.063
Level 6	31,238	312	99.88		0.69	(0.61–0.77)	<0.001
Level 7	28,404	247	86.96		0.62	(0.55–0.71)	<0.001
DCSI score ^a^				<0.001			
0	442,189	3929	88.85		1		
1	155,131	1729	111.45		1.10	(1.04–1.17)	<0.001
≥2	131,383	1859	141.49		1.20	(1.13–1.27)	<0.001
Hyperlipidemia				<0.001			
No	574,597	5,802	100.98		1		
Yes	154,106	1715	111.29		1.03	(0.97–1.08)	0.383
Hyperuricemia				0.696			
No	722,413	7,449	103.11		1		
Yes	6,290	68	108.11		0.93	(0.74–1.19)	0.577
Cerebrovascular disease				<0.001			
No	690,054	6,926	100.37		1		
Yes	38,649	591	152.91		0.97	(0.89–1.06)	0.524
Obesity				0.039			
No	725,531	7,496	103.32		1		
Yes	3,172	21	66.20		0.75	(0.49–1.15)	0.189
Alcoholism				0.463			
No	728,262	7,514	103.18		1		
Yes	441	3	60.23		0.87	(0.28–2.71)	0.816
NAFLD ^a^				0.293			
No	722,530	7,445	103.04		1		
Yes	6,173	72	116.64		1.17	(0.93–1.47)	0.192
RA ^a^				0.109			
No	722,471	7,440	102.98		1		
Yes	6,232	77	123.56		1.07	(0.85–1.34)	0.560
Hypothyroidism				<0.001			
No	725,490	7,465	102.90		1		
Yes	3,213	52	161.84		1.47	(1.12–1.93)	0.006
HBV ^a^				0.773			
No	728,574	7,514	103.16		1		
Yes	129	3	77.52		0.88	(0.12–6.27)	0.901
HCV ^a^				0.013			
No	725,443	7,469	102.96		1		
Yes	3,260	48	147.24		1.33	(0.99–1.77)	0.051
Sleep disturbance				<0.001			
No	569,717	5,534	97.14		1		
Yes	158,986	1983	124.73		1.08	(1.02–1.13)	0.007
SLE ^a^				0.125			
No	728,319	7,510	103.11		1		
Yes	384	7	182.29		1.77	(0.84–3.72)	0.130
CKD ^a^				<0.001			
No	722,880	7,427	102.74		1		
Yes	5,823	90	154.56		0.96	(0.77–1.18)	0.689
Migraine				0.203			
No	725,088	7,472	103.05		1		
Yes	3,615	45	124.48		1.28	(0.96–1.72)	0.098
Hyperthyroidism				0.681			
No	724,367	7,475	103.19		1		
Yes	4,336	42	96.86		1.00	(0.74–1.35)	0.982

^a^ Abbreviations: DCSI, diabetes complications severity index; NAFLD, non-alcoholic fatty liver disease; RA, rheumatoid arthritis; HBV, hepatitis B virus; HCV, hepatitis C virus; SLE, systemic lupus erythematosus; CKD, chronic kidney disease.

After adjusting for the relevant variables containing sex, age, income level, urbanization, DCSI, and AMD-related comorbidities, we determined that patients with DM treated with metformin at <5, 5–15, 15–25, and >25 DDD/month for AMD had ORs of 0.93 (95% CI: 0.88–0.99), 1.00 (95% CI: 0.95-1.07), 1.01 (95% CI: 0.91-1.12), and 1.39 (95% CI: 1.08-1.78), respectively. Patients aged ≥75 years had an OR of 6.40 (95% CI: 5.82-7.05) compared to patients aged 50–54 years. Patients with a DCSI score of 2 had a higher risk of AMD (OR: 1.20, 95% CI: 1.13-1.27). Moreover, Patients with comorbid hypothyroidism (OR: 1.47, 95% CI: 1.12-1.93), sleep disturbance (OR: 1.08, 95% CI: 1.02-1.13) had a higher risk of AMD at 5-year follow-up. However, patients with comorbid hyperlipidemia, hyperuricemia, CVD, obesity, alcoholism, NAFLD, RA, HBV, HCV, SLE, CKD, migraine, or hyperthyroidism did not exhibit a notable risk of AMD.

## Discussion

This study found that treatment with metformin may be associated with the risk of AMD among patients with T2DM in a dose-response relationship manner. The results suggest that the intensity of treatment with metformin <5 DDD/month is associated with a lower risk of AMD at 5 years after initial DM diagnosis. However, patients with T2DM treated with >25 DDD/month of metformin experienced higher risks of AMD at 5 years. In addition, we found that among patients T2DM treated with metformin, older patients and patients with a higher DCSI score had a higher risk of AMD. Furthermore, patients with T2DM with comorbid sleep disturbance and hypothyroidism had a higher risk of AMD.

DM may play a significant role in the progression and development of AMD. Previous studies have demonstrated a positive correlation between DM and AMD (Nitsch et al., 2008; Topouzis et al., 2009; Choi et al., 2011; Chen et al., 2014; Khotcharrat et al., 2015; Vassilev et al., 2015; He et al., 2018). Several pathophysiological mechanisms may be associated with DM and AMD. Oxidative stress and chronic inflammation may explain the correlation between DM and the risk of AMD. Oxidative stress causes outer blood–retinal barrier degeneration that contributes to AMD progression (Jung et al., 2022), and oxidative stress is a risk factor for the development of insulin resistance through insulin signal disruption (Houstis et al., 2006; Newsholme et al., 2019).

Metformin achieves its antioxidative and anti-inflammatory effects through the activation of AMP-activated protein kinase (AMPK) (Lee et al., 2013; Zhao et al., 2020) and reduction in reactive oxygen species (Hou et al., 2010). Recent studies have demonstrated that AMPK plays a major role in the regulation of systemic glucose homeostasis and metabolic stress. AMPK is a conserved energy sensor and master regulator of glucose metabolism, which restores cellular energy balance during metabolic stress (Garcia and Shaw, 2017) and might be involved in AMD pathogenesis (Brown et al., 2019a). Metformin inhibited oxidative stress on human retinal pigment epithelium (RPE) cells by stimulating the AMPK signaling pathway in a mouse model of AMD (Xu et al., 2018). Antioxidant and anti-inflammatory effects of metformin can protect the RPE cells against the lesions of early AMD (Jiang et al., 2022).

Our findings demonstrated that patients with T2DM treated with <5 DDD/month of metformin had a lower risk of AMD at 5 years after initial DM diagnosis. Animal studies and physiology studies have suggested that metformin may play a beneficial role in the prophylaxis of AMD (Amin et al., 2022). Several studies suggested that metformin may have a role in AMD development and progression (Romdhoniyyah et al., 2021; Chen et al., 2019; Blitzer et al., 2021). A large-scale study reported the protective outcomes of metformin use in the development of AMD, with a 42% reduction (Brown et al., 2019b). A systematic review and meta-analysis study found that treatment with metformin is not associated with a significant lower risk of AMD (Romdhoniyyah et al., 2021). Another large case-control study reported that treatment with metformin is associated with a lower risk of AMD, with the lowest ORs associated with low-to-moderate doses (Blitzer et al., 2021). This study suggests that metformin use more than 2 years in patients aged 55 years and older is correlated with 5%–10% decreased odds ratio of AMD development.

Our findings revealed that patients treated with >25 DDD/month of metformin exhibited a higher risk of AMD after 5 years of follow-up. A case–control study observed no significant associations between AMD risk and cumulative duration or exposure of treatment with metformin (Lee et al., 2019). Another study with a small sample size found a conflicting relationship between metformin exposure and dry AMD, with the findings based on assessment of metformin cumulative dosage and the intensity of the treatment with metformin (Eton et al., 2022). A study based on medical claims from a large US insurer also indicated that conflicting associations between metformin exposure and development of dry AMD. Cumulative metformin dosage model showed a significant association between the risk of dry AMD with cumulative dosage, with the lowest dosage quartile associated with a decreased risk of dry AMD and the highest dosage associated with an increased risk (Eton et al., 2022). Active treatment with metformin is associated with an increased risk of dry AMD, whereas prior treatment with metformin is associated with decreased risk (Eton et al., 2022). Our findings are similar to a large nationwide case-control study revealed that the use of metformin may protect against AMD development in a dose-dependent manner (Blitzer et al., 2021). This research found that metformin may be useful as a preventive treatment for AMD with strongest at low to moderate doses, while higher dose did not have reduced risk of AMD development. This study reported that doses of greater than 1080 g of metformin use more than 2 years did not have decreased risk of AMD development, while was particularly for low to moderate doses of metformin revealed the greatest potential benefit (Blitzer et al., 2021). The greatest reduction in AMD risk was observed at metformin doses of 271–600 g over 2 years with an OR of 0.91, and doses of 1–270 g and 600–1080 g over 2 years were also correlated with decreased OR, 0.93 and 0.95, respectively (Blitzer et al., 2021).

Vitamin B12 deficiency may play a role in AMD development in patients with T2DM receiving long-term treatment with metformin. Treatment with metformin can induce vitamin B12 malabsorption by increasing bacterial overgrowth, altering gut bacterial flora in the enteric canal, and binding to the vitamin B12 intrinsic factor (Zhang et al., 2016). Malabsorption contributes to a decreased serum vitamin B12 plasma level. Current evidence suggests that metformin impairs vitamin B12 status in a dose-dependent and duration-dependent association manner (Infante et al., 2021). A meta-analysis suggest a negative association between metformin use and vitamin B12 plasma levels in T2DM patients (Chapman et al., 2016), and higher cumulative exposure and longer duration of metformin treatment were associated with an increased risk of vitamin B12 deficiency (Khattar et al., 2016; Huang et al., 2022a; Huang et al., 2022b; Huang et al., 2023). Patients received metformin with therapy duration ≥ 5 years and a metformin dose of ≥ 1500 mg/day for a duration of at least 6 month was associated vitamin B12 deficiency, especially the highest risk has been found in patients with a daily metformin dose of ≥ 2000 mg (Infante et al., 2021). T2DM patients received metformin dosage of >2,000 mg/day increased the risk of vitamin B12 deficiency 22 times (Ko et al., 2014). However, the underlying mechanism accounting for metformin-induced vitamin B12 deficiency in patients with long-term and high-dose of metformin use remains unclear. Nevertheless, the proposed underlying mechanisms due to the alteration in small intestine motility, resulting in small intestinal bacterial overgrowth and subsequent B12 deficiency or by directly decreasing vitamin B12 absorption (Ting et al., 2006; Damiao et al., 2016); malabsorption leads to a decreased serum vitamin B12 level. Vitamin B12 and homocysteine may play a role in reducing the risk of AMD. Vitamin B12 deficiencies, folate, or elevated serum homocysteine levels were used as predictors of a high risk of AMD (Gopinath et al., 2013). Vitamin B12 is essential for the conversion of homocysteine to methionine in the methionine cycle (Allen, 2012). Vitamin B12 deficiency can impair the remethylation of homocysteine; moreover, metformin-induced vitamin B12 deficiency is potentially associated with hyperhomocysteinemia (Russo et al., 2011). An animal study found that excess homocysteine levels on the structure and function of retinal pigment epithelial that contribute to the development of AMD-like features (Ibrahim et al., 2016). Human study have reported that plasma homocysteine level was elevated in patients with AMD and highlighted a strong correlation between hyperhomocysteinemia and the development of AMD (Huang et al., 2015). A cross-sectional study found that increased total serum homocysteine and low vitamin B12 concentrations were independently associated with a higher risk of AMD (Rochtchina et al., 2007). The beneficial effects of vitamin B12 and folate on the risk of AMD are partly mediated by lowering the concentration of serum homocysteine (Rochtchina et al., 2007). Although treatment with metformin can decrease the risk of AMD (Brown et al., 2019a; Xu et al., 2018; Jiang et al., 2022), when long-term and high-dose or high cumulative dosage of metformin use were associated with biochemical B12 deficiency and hyperhomocysteinemia (Russo et al., 2011), may offset the protection effect of metformin and could lead to enhance the risk of AMD (Rochtchina et al., 2007). Routine assessment of vitamin B12 levels in individuals treated with metformin should be considered (Aroda et al., 2016; Al-Hamdi et al., 2020). Due to the clinical benefits of metformin use, its associated side effects such as metformin-induced vitamin B12 deficiency is often overlooked in T2DM patients. However, the diagnosis of metformin-induced vitamin B12 deficiency may be difficult (Al-Hamdi et al., 2020). The underlying mechanisms of metformin cumulative dosage and the risk of AMD remain unclear. Thus, further prospective clinical trials are warranted to investigate the protective effect of metformin on AMD, especially regarding duration and dosage of therapy.

Our findings showed that T2DM patients treated with metformin, older patients, and having a higher DCSI score linked to an increased risk of AMD. Previous studies have identified several risk factors for AMD, including aging, ocular disorders, systemic diseases, smoking, diet, genetic susceptibility, and environmental risk factors (Lim et al., 2012), with aging being the strongest risk factor (Aldebert et al., 2018). In the general population, vitamin B12 plasma levels decline with age, and thus, the prevalence of vitamin B12 deficiency increases with age. Age is a strong risk factor for the development of AMD, and individuals aged <50 years have a lower risk of AMD (Jiang et al., 2022) compared with older adults, who also have a higher risk of vitamin B12 deficiency (Gonzalez-Gross et al., 2007). The DCSI is a useful tool for adjusting for baseline severity of disease and predicting mortality and the risk of hospitalization among patients with DM (Young et al., 2008). Our study showed that patients with T2DM treated with metformin with higher DCSI scores had an increased risk of AMD. Thus, DCSI may be used as an indicator for assessing the risk of AMD development.

Our study results demonstrated that patients with T2DM treated with metformin and with comorbid sleep disturbance and hypothyroidism had a higher risk of AMD. A Taiwan population-based study indicated that insomnia is an independent indicator of an increased risk of AMD (Tsai et al., 2020). Thyroid disease is associated with an increased risk of AMD (Xu et al., 2021).

This study adopted a population-based design and used data from the NHIRD. Because we included the entire Taiwanese population in this study, the sample size is large and sufficient for reducing selection bias and providing high-quality data. Second, the characteristics of the database provide sufficient statistical power for investigating the association between treatment with metformin and the risk of AMD among patients with T2DM. Finally, the intensity of treatment with metformin (DDD/month) was <5, 5–15, 15–25, >25 for determining the relationship between patients with T2DM and the risk of AMD.

This population-based cohort study has several limitations. First, information regarding family histories of AMD among patients with T2DM was unavailable. Second, patients’ personal data and their lifestyle habits, such as body mass index, cigarette smoking habits, alcohol consumption, dietary habits, and physical activity (factors that are associated with AMD risk), were unavailable. Due to the limitations of the Taiwan National Health Insurance inpatient medical claims system, the information on the medication dosage during hospitalization was lacking from the NHIRD. Therefore, the use of metformin during hospitalization was not included in the present study, which may result in an underestimation of metformin’s DDD in our study. Third, the diagnoses of AMD and other comorbidities were coded in accordance with the *ICD-9-CM* and *ICD-10-CM*. Nonetheless, the NHI Bureau of Taiwan randomly reviews the charts and interviews patients to assess the accuracy of the diagnoses, which improves the accuracy and validity of the NHIRD. Fourth, Information regarding biochemical parameters (e.g., fasting glucose, HbA1C, urine protein) is unavailable in the database but may affect developing AMD factors. The severity of DM and the disease duration of DM may also affect developing AMD. Therefore, the present study enrolled the new-onset DM patients as the study subjects and used the DCSI to adjust the severity of DM to reduce the bias. This study was a nationwide population-based study. Thus, the study results have accuracy and representativeness. Finally, this study is a type of epidemiology observational study that analyzes data from a nationwide database. The study result can only provide evidence to demonstrate that metformin is related to incident AMD. It is essential to obtain more information from other databases or questionnaires to conduct a prospective study or randomized controlled trial to analyze the cause-effect relation in future research.

## Conclusion

In conclusion, this study provides evidence that treatment with metformin may be associated with the risk of AMD among patients with T2DM in a dose-dependent association manner. Patients treated with <5 DDD/month of metformin had a decreased risk of AMD at 5 years. However, >25 DDD/month exhibited an increased risk of AMD.

## Data Availability

The database used to support the findings of this study was provided by the Health and Welfare Data Science Center, Ministry of Health and Welfare (HWDC, MOHW) under license and so cannot be made freely available. Requests for access to these data should be made to HWDC (https://dep.mohw.gov.tw/dos/cp-5119-59201-113.html).
